# The Impact of Cell-Free DNA Analysis on the Management of Retinoblastoma

**DOI:** 10.3390/cancers13071570

**Published:** 2021-03-29

**Authors:** Amy Gerrish, Helen Jenkinson, Trevor Cole

**Affiliations:** 1 West Midlands Regional Genetics Service, Birmingham Women’s and Children’s NHS Foundation Trust, Birmingham B15 2TG, UK; trevor.cole1@nhs.net; 2 Department of Paediatric Oncology, Birmingham Women’s and Children’s NHS Foundation Trust, Birmingham B4 6NH, UK; hjenkinson1@nhs.net

**Keywords:** retinoblastoma, cell-free DNA, liquid biopsy, noninvasive prenatal diagnosis

## Abstract

**Simple Summary:**

Retinoblastoma is a childhood eye cancer caused almost entirely by defects in a gene known as *RB1*. Other genetic changes within the tumour are also thought to affect the progression of disease. Until recently, tumour DNA could only be analysed if the eye was removed as part of patient treatment. However, recent research has shown that the analysis of a particular type of DNA, known as cell-free DNA, within the eye fluid or blood of patients, can be used to detect changes in the *RB1* gene or other parts of the genome within a retinoblastoma tumour. The analysis of cell-free DNA in the blood of pregnant women can also be used to detect whether the unborn baby will be affected with retinoblastoma. In this review, we summarise these studies and discuss the potential impact of cell-free DNA analysis on retinoblastoma patient management in the future.

**Abstract:**

Retinoblastoma is a childhood eye cancer, mainly caused by mutations in the *RB1* gene, which can be somatic or constitutional. Unlike many other cancers, tumour biopsies are not performed due to the risk of tumour dissemination. As a result, until recently, somatic genetic analysis was only possible if an affected eye was removed as part of a treatment. Several recent proof of principle studies have demonstrated that the analysis of tumour-derived cell-free DNA, either obtained from ocular fluid or blood plasma, has the potential to advance the diagnosis and influence the prognosis of retinoblastoma patients. It has been shown that a confirmed diagnosis is possible in retinoblastoma patients undergoing conservative treatment. In vivo genetic analysis of retinoblastoma tumours is also now possible, allowing the potential identification of secondary genetic events as prognostic biomarkers. In addition, noninvasive prenatal diagnosis in children at risk of inheriting retinoblastoma has been developed. Here, we review the current literature and discuss the potential impact of cell-free DNA analysis on both the diagnosis and treatment of retinoblastoma patients and their families.

## 1. Introduction

Retinoblastoma is a childhood intraocular cancer. It can present in a unilateral form, where the disease develops in a single eye, or bilateral, where both eyes are affected. The age of onset is usually less than three years old, and for patients with bilateral disease, it is often significantly earlier, before 12 months of age [1]. The incidence rate has been calculated at 1:15–20,000 [2], with approximately 45 children diagnosed annually in the UK [3], where the population is 66 million. While retinoblastoma is lethal if left untreated, it is considered highly curable in countries where early detection and multiple treatment modalities are available, resulting in disease-free survival rates over 97% [4]. In middle- and lower-income countries this figure is significantly reduced, leading to an average global patient survival rate of less than 30% [5].

### 1.1. Genetics of Retinoblastoma

Over 99% of retinoblastomas are due to the inactivation of the *RB1* tumour suppressor gene, caused by either two somatic mutations or an initial germline mutation followed by a subsequent somatic hit [1]. Individuals who carry a constitutional mutation are at risk of developing bilateral disease, as well as nonocular second malignancies later in life, including osteosarcomas and soft-tissue sarcomas [6]. Siblings of germline carriers are also at an increased risk of inheriting the *RB1* gene mutation, from either a heterozygous or mosaic parental carrier. Therefore, identifying the genetic cause of retinoblastoma is important for planning the management of the retinoblastoma itself and the possible nonocular second primary cancers of affected individuals, as well as determining the disease risk in their extended family.

Germline *RB1* variants can comprise single-nucleotide variants (SNVs) and indels, as well as copy number variation (CNVs). Somatic *RB1* mutations can consist of these types of genetic change, as well as a loss of heterozygosity (LOH) and hypermethylation of the promoter [7,8]. To detect this spectrum of variation, current molecular diagnostic testing analyzes both germline DNA and tumour DNA (where available) and requires a combination of technologies that can include next-generation sequencing (NGS), microarray, multiplex ligation-dependent probe amplification (MLPA), LOH detection and promoter methylation analysis [2]. A small minority of retinoblastoma cases (<1%) have been found to have two active copies of the *RB1* gene but somatic *MYCN* amplification. These patients have an aggressive form of the disease with an age of onset of less than six months [9].

While retinoblastomas are initiated by genetic alterations in *RB1* or, rarely, *MYCN*, additional somatic changes have been identified that are thought to further drive tumourgenesis. These appear to be mostly limited to CNVs and include gains of 1q, 2p, 6p, 7q, and 19p, as well as losses of 13q and 16q [10], although recurrent single-nucleotide variation has been observed in *BCOR* and *CREEBP* [11,12]. The most commonly reported CNVs are gains in 1q and 6p, observed in over 40% of retinoblastoma tumours [13].

### 1.2. The Impact of Retinoblastoma Treatment Advances On Genetic Testing

Despite an excellent prognosis for many decades, ocular salvage was limited until the late 20th century. External beam radiotherapy or the immediate removal of an eye, known as primary enucleation, were often the only options open to ophthalmologists following a diagnosis of retinoblastoma [14]. Although first described in the 1950s [15], it was not until the early 1990s that multi-agent intravenous chemotherapy was widely adopted for the treatment of retinoblastoma. Since then, newer techniques have been developed that aim to not only cure the disease but, also, conserve the eye and retain vision, whilst minimising a child’s exposure to systemic chemotherapy, which is associated with potential long-term morbidity. These treatments include intra-arterial (IAC) and intra-vitreal (IViC) chemotherapy, often combined with localised treatments such as thermotherapy and cryotherapy [16]. If conservative treatment is unsuccessful, a secondary enucleation may be required. Nevertheless, overall enucleation rates have significantly reduced, from over 95% to less than 10%, in the last 15 years [14]. While this decline in enucleation rates represents a great improvement in patient outcome, a challenging but inevitable consequence of increased eye conservation is a lack of tumour-derived DNA for somatic studies. Undertaking a biopsy of an intraocular retinoblastoma is rarely indicated, as most cases can be confidently diagnosed without tissue confirmation, and there is a small but discernable risk of tumour dissemination [16,17]. As a result, if an eye is not removed, tumour DNA cannot be accessed, and somatic variants cannot not be identified. Due to the fact that current molecular diagnostic testing is less than 100% sensitive [2] and the low but identifiable risk of germline mosaicism [18], a nonhereditary status cannot be assigned solely from a negative germline screening result. The identification of both somatic variants and subsequent exclusion of these variants from the germline is required. Consequently, a definitive nonheritable diagnosis is currently not possible for unilateral patients who have had successful conservative treatment, even when no germline *RB1* mutation has been detected. Therefore, these patients continue to need ocular screening for retinoblastoma in their unaffected eye, typically under anaesthetic. They also require lifelong follow-up due to the possible risk of additional nonocular second malignancies, conferred by a germline *RB1* mutation. A lack of a definitive diagnosis significantly increases the psychological impact of the disease on families [19,20]—in particular, the uncertainty around the risk of second primary cancers later in life. Moreover, when the underlying genetic aetiology remains unknown, any offspring and siblings of these patients will undergo ocular screening until at least the age of three, unless the risk is further reduced in the latter individuals by linkage exclusion studies [21]. As well as creating an additional burden on the families of retinoblastoma sufferers, a lack of definitive diagnosis has also been shown to significantly increase healthcare costs [22].

An absence of biopsy material has also meant that in vivo somatic molecular data from patients undergoing eye salvage treatment has not previously been available. An increasing number of clinical trials are using tumour molecular genetic information for the accurate prognostication and precision treatment for a wide range of cancers [23]. However, to date, the inability to obtain such data for cases of retinoblastoma without enucleation has severely restricted their clinical utility and, as a consequence, been a barrier to research that could inform the prognosis or best treatment options for these patients.

### 1.3. Clinical Application of Cell-Free DNA Analysis

Fragmented, extracellular DNA was first detected in the human circulatory system in 1948 [24]. Subsequent research into the origin of this cell-free DNA (cfDNA) has found it to be a complex process involving cellular breakdown mechanisms such as apoptosis and necrosis, as well as active release [25]. The clinical importance of cfDNA was initially proposed after significant increases in cfDNA levels were observed in patients with autoimmune disease and other disorders, including cancer [26,27]. Following this, Stroun and colleagues were able to demonstrate that a fraction of the cfDNA in cancer patients originates from cancer cells [28], leading to the hypothesis that the analysis of cfDNA and, more specifically, circulating tumour DNA could be an alternative to solid tumour biopsy. With the recent advent of more sophisticated technologies to isolate and analyse cfDNA, research into the potential of liquid biopsies has intensified [29]. The utility of which has been proposed in multiple areas of cancer patient management, including diagnosis, staging, and prognosis, as well as monitoring of the treatment response [30].

**Figure 1:**
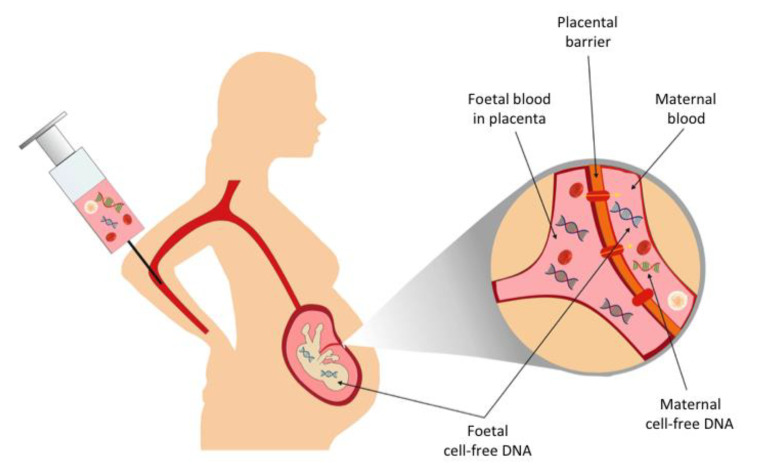
Noninvasive prenatal diagnosis (NIPD). The blood of a pregnant woman contains both maternal and foetal-derived cell-free DNA. Genetic analysis of the cell-free foetal DNA by NIPD can determine whether a fetus has inherited a pathogenic variant. Variants can be detected directly, or an indirect analysis, such as the relative haplotype dosage (RHDO) analysis, can be used.

A further clinical application for the analysis of cfDNA was indicated when foetal-derived cfDNA was detected in the blood of pregnant women [31]. A relatively high foetal fraction [32], combined with a short half-life [33], has lead to the utilisation of cfDNA analysis in noninvasive prenatal testing, including sex determination, Rhesus status, and aneuploidy [34]. More recently, noninvasive prenatal diagnosis (NIPD) for single gene disorders has been developed [35]. NIPD analysis can be performed on DNA extracted from maternal blood taken as early as eight weeks gestation (Figure 1). As invasive prenatal testing via chorionic villus sampling (CVS) or amniocentesis is only available from 11 and 16 weeks, respectively, NIPD has the advantage of an earlier diagnosis, as well as an absence of the reported 0.5% risk of miscarriage associated with invasive prenatal tests [36,37].

The application of both liquid biopsy and noninvasive prenatal diagnosis offers a wealth of new possibilities to retinoblastoma patients, and significant research has been published in this area in recent years (Table 1). In this review, we summarise these findings and outline the potential of cell-free DNA to advance both retinoblastoma diagnosis and prognosis and, as a result, patient management.

## 2. Diagnosis of Retinoblastoma

### 2.1. Diagnosis Using Aqueous Humour

While a tumour biopsy is not feasible during conservative treatment of retinoblastoma, a sample of the eye fluid, aqueous humour, is obtained during intra-vitreal chemotherapy (IViC). IViC is used to treat vitreous seeding through the injection of either melphalan or melphalan combined with topotecan into the posterior chamber. In order to equalise the ocular pressure, approximately 100 µL of aqueous humour is removed from the eye prior to chemotherapy injection. IViC has become a widely implemented treatment choice following the development of this enhanced protocol [49], where extraocular dissemination of the tumour was found to be highly unlikely [50].

**Figure 2:**
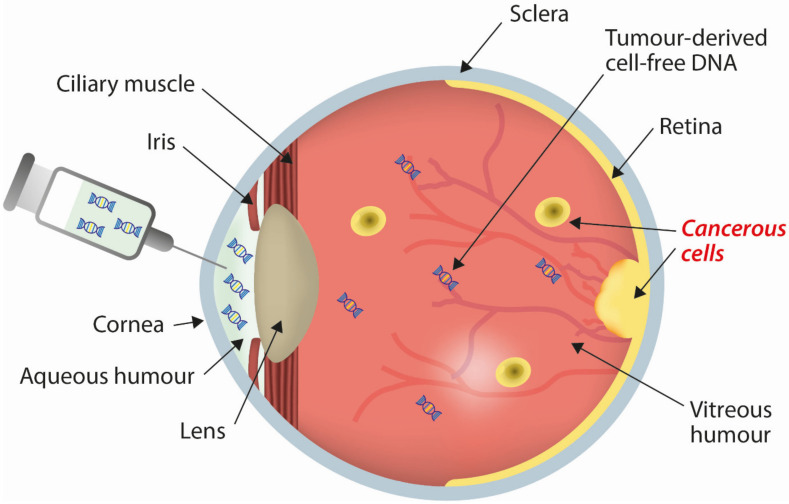
Sampling cell-free DNA in aqueous humour from a retinoblastoma eye. Tumour-derived cell-free DNA is present in the vitreous and aqueous humour. Approximately 100 µL of aqueous humour is collected using a 32-guage needle.

While aqueous humour aspirated during IViC has often been routinely discarded as a waste product, its clinical potential has previously been recognised. Both nucleic acids and proteins have been detected within aqueous humour from retinoblastoma patients [51], as well as patients with other ocular diseases [52,53]. Therefore, we and others hypothesised that aqueous humour could be a source of circulating tumour DNA and, as a result, used as a surrogate tumour biopsy for patients undergoing conservative treatment (Figure 2). While only a small volume (less than 100 μL) of aqueous humour is obtained, the proximity to the tumour, the closed nature of the eye, and the relative low turnover of fluid within the compartment [54] suggested that the levels of tumour-derived cfDNA could be sufficient for a genetic analysis.

In 2017, measurable levels of cell-free DNA were detected in aqueous humour samples taken from retinoblastoma patients [40]. Moreover, the genetic profiling of this cfDNA suggested it was derived from the tumour. Berry et al. [40] performed shallow whole-genome sequencing (WGS) and CNV profiling on six aqueous humour samples obtained from the eyes of retinoblastoma patients. This included two samples taken during the primary enucleation, with the remaining samples obtained from a single patient during three sequential IViC treatments and the subsequent secondary enucleation. Highly correlated CNV profiles, indicative of a secondary somatic events, were observed between the aqueous humour-derived cfDNA and the paired tumour DNA samples, implying that the genetic analysis of aqueous humour could be utilised for somatic profiling where tumour tissue is unavailable.

In 2019, we also reported on cfDNA analysis in retinoblastoma and, for the first time, described the detection of somatic *RB1* pathogenic variants in aqueous humour [38]. We analysed DNA from 10 retinoblastoma patients who underwent an eye enucleation as part of their treatment. Routine clinical samples of tumour DNA, extracted from the tumour biopsy post enucleation, along with genomic DNA were compared against cfDNA extracted from an aqueous humour sample taken prior to opening the eye for histopathological examination. Following targeted capture-based next-generation sequencing (NGS) of the *RB1* region, we were able to detect both *RB1* pathogenic mutations in all 10 cfDNA samples, 18 of which were somatic and two were germline mosaic. The variants comprised 11 SNVs, two CNVs, and seven regions of LOH that spanned the *RB1* gene. Interestingly, mutant allele frequencies suggested that the majority of cfDNA within aqueous humour is tumour-derived. This finding was supported by the observation that cfDNA from aqueous humour had a smaller fragment size profile than that of cfDNA derived from plasma. Several publications have previously shown that tumour-derived cfDNA is shorter than that of circulating nuclear DNA [55,56]. In addition to the identification of *RB1* mutations within aqueous humour obtained from enucleated eyes, we were also able to detect both *RB1* pathogenic variants in aqueous humour taken from two patients undergoing IViC. As these patients were undergoing conservative treatment, no tumour DNA was available, and the somatic variants identified were previously unknown.

While these two studies show proof of principle that a genetic analysis of aqueous humour-derived cfDNA could be used as a surrogate tumour biopsy in the future, it should be noted that the levels of cfDNA we observed within aqueous humour taken during IViC were markedly lower than those found in enucleated eyes (<0.1 ng/µL vs. 87 ng/µL). This is perhaps unsurprising, given that the majority of cfDNA in aqueous humour appears to be tumour-derived and patients undergoing IViC have significantly reduced tumour burden compared to those patients whose eyes require primary enucleation. This finding is supported by the work of an independent group [48] and by our own additional observation that the cfDNA levels in aqueous humour taken from secondary enucleations are also markedly reduced compared to eyes removed immediately upon diagnosis [38]. It is therefore likely that, for this assay to be implemented clinically, aqueous humour samples taken at or soon after diagnosis will be required to produce a robust clinical assay.

### 2.2. Diagnosis Using Plasma

While a sample of aqueous humour is obtained during IViC treatment, this type of therapy is not performed in all patients undergoing conservative management [5]. Furthermore, as outlined above, data from ourselves and others [38,48] suggest so-called “diagnostic taps”, aqueous humour samples taken much earlier in the treatment process, will be required in order to fully utilise the potential of this assay. While the extraction of aqueous humour is a minimally invasive procedure with a low risk of complication [50], a liquid biopsy using cfDNA taken from blood has several advantages over an aqueous humour assay, including a less-invasive sampling procedure and the provision of a larger sample volume.

A recent publication used targeted capture-based NGS to analyse tumour DNA, genomic DNA, and plasma-derived cfDNA from 10 unilateral retinoblastoma patients who had advanced intraocular disease, three which went on to develop metastatic disease [47]. Plasma cfDNA was analysed using capture-based NGS targeted to the *RB1* gene. *RB1* pathogenic variants were identified in tumour and genomic DNA using MSK-IMPACT, a NGS panel that targets over 400 genes associated with cancer, including all coding exons of *RB1* [57]. Analysis of the plasma-derived cfDNA, blind to the tumour results, identified seven *RB1* SNV mutations, which were subsequently confirmed in the tumour. Six of these variants were identified with a frequency of 1–20%; the remaining SNV was detected at 0.8%. In one of the 10 patients analysed, both somatic *RB1* variants were identified, providing a proof of principle that plasma-derived cfDNA could potentially be used for diagnostic liquid biopsy. However, the seven identified mutations represented only half of those identified in the matching tumour sample (*n* = 13). Furthermore, two additional *RB1* mutations were detected at a frequency of 1.56% and 0.99% within the patient cohort that were not detected in the paired tumour DNA. While the authors suggest these mutations could be derived from a subpopulation of cells in the eye, they could not rule out the possibility of false positives. Complementary to the work performed on aqueous humour, Kothari et al. [47] also reported that tumour-derived cfDNA levels within the plasma appear to decrease even after just one cycle of chemotherapy. Based on these findings, a larger study is therefore required, including individuals in earlier stages of disease, along with pretreatment sampling, to further investigate the possibility of a diagnostic assay for retinoblastoma using cell-free DNA derived from plasma.

### 2.3. Noninvasive Prenatal Diagnosis

A child is at 50% risk of inheriting a *RB1* mutation if either of their parents carries a germline *RB1* variant. Furthermore, siblings of a patient with an apparent de novo mutation have an approximate 1% risk of also carrying the mutation due to germ cell mosaicism [58]. Where a family history is known, early diagnosis is paramount to achieve an optimal clinical outcome with the least treatment morbidity. Currently, the majority of diagnostic testing is performed on postnatal cord blood. There are many logistical and psychological disadvantages to this type of testing, including the challenges in obtaining a cord blood and urgent result from a geographically distant patient at a time when family bonding and psychological well-being is a priority [59,60,61]. Invasive prenatal testing is available to parents, but uptake is generally low, at less than 12% [62].

The analysis of fetus-derived cfDNA through a noninvasive prenatal diagnosis (NIPD) has recently been clinically implemented for several monogenetic disorders [63]. Using a combination of NGS techniques, depending on the family history, we developed NIPD for retinoblastoma [39] that offers significant advantages over both newborn and invasive prenatal testing. Testing is available for both paternally and maternally inherited *RB1* mutations, as well as suspected de novo variants, where the parents are unaffected but a previous child carries a germline *RB1* mutation. Paternal and de novo variants are detected directly through amplicon-based NGS. However, the direct detection of maternally inherited pathogenic variants is challenging due to the presence of cfDNA of maternal original within maternal plasma. To overcome this, we used capture-based NGS, targeted to the *RB1* region, and relative haplotype dosage analysis (RHDO). In this type of analysis, the maternal haplotypes inherited by the fetus are compared to the haplotypes inherited by a previous child. The previous child’s *RB1* gene status (heterozygous mutation carrier or nonmutation carrier) can be assigned to one of the two maternal chromosomes. The relative haplotype dosage of cfDNA can then be used to determine whether the fetus carries the maternal pathogenic *RB1* mutation. While haplotype phasing avoids the technical challenges of directly analysing a maternally inherited variant, the requirement of a previous child is a significant disadvantage of RHDO. In the future, an alternative option for primigravida maternal *RB1* carriers may be the use of maternal parental (foetal grandparental) DNA for haplotype phasing in those families with two prior affected generations [64,65]. Another possibility is microfluidics-based link-read sequencing, which allows the direct phasing of parental haplotypes without the need for a reference sample [66,67].

## 3. Prognosis of Retinoblastoma

Several studies have previously attempted to identify putative prognostic biomarkers for retinoblastoma in both aqueous humour [51] and blood [68]. However, the routine detection of circulating tumour DNA and the development of liquid biopsies for other cancers [69] has increased the possibility for the future prediction and monitoring of treatment response in retinoblastoma patients.

### 3.1. Prognosis Using Aqueous Humour

In a series of publications, Berry and colleagues [40,41,42] examined the potential of analysing cfDNA within aqueous humour to detect somatic copy number alterations (SCNAs). In their proof of principle study, described in Section 2.1, highly correlated CNV profiles were observed between the aqueous humour-derived cfDNA and paired tumour DNA samples, implying that the genetic analysis of aqueous humour could be utilised for the profiling of secondary somatic events in vivo [40].

In a follow-up publication [41], CNV profiling was performed on an extended cohort of 63 aqueous humour samples from 26 retinoblastoma patients (29 eyes), the majority of which were taken during active treatment. Sixteen eyes required enucleation, while the other 13 eyes were saved. SCNAs were found in two-thirds of aqueous humour samples, 80% of which were SCNAs previously shown to be highly recurrent in retinoblastoma tumours, namely gains of 1q, 2p (including focal MYCN amplification), and 6p or a loss of 13q or 16q [10]. Moreover, when the presence of these highly recurrent retinoblastoma SCNAs was correlated with the clinical outcome, significantly more were observed in aqueous humour from eyes requiring enucleation (>90%) compared to eyes that were saved (<40%). Furthermore, by analysing serial aqueous humour samples from two patients, the authors found SCNA amplitude correlated with clinical response. A sequential decrease in the magnitude of several SCNAs was observed during the treatment of one patient where the eye was subsequently salvaged. In contrast, an increase or stability of SCNA amplitude was observed in aqueous humour taken from an eye with persistent tumour activity, which was later enucleated.

When the recurrent retinoblastoma SCNAs were analysed as individual regions, the association appeared to be mainly driven by a gain in chromosome 6p, the presence of which was associated with a 10-fold increase in the chance of enucleation. Moreover, the inclusion of the presence of 6p amplification with clinical classification (either International Intraocular Retinoblastoma Classification (IIRc) or the American Joint Committee on Cancer TNM scheme) increased the predictive value of a tumour relapse requiring enucleation over classification alone. A concordance between the tumour and aqueous humour was found to be >90% in all patients except in eyes where multifocal tumours were present, indicating that the analysis of aqueous humour could provide a combined multiclonal analysis of a retinoblastoma tumour.

These findings—in particular, the association between a gain of 6p and the likelihood of enucleation—remained consistent in an increased cohort of 50 patients [42], strengthening the potential of 6p amplification as a prognostic biomarker for a lack of treatment response. In addition, Xu et al. [42] observed that the amplitude of a 6p gain was significantly greater in enucleated eyes compared to those that were salvaged. The group further investigated this in 20 patients where serial aqueous humour samples were available [44]. As well as performing their original SCNA amplitude analysis, a tumour fraction was determined using icorCNA software, which calculates the tumour fraction of a given sample using somatic SNV or copy number changes detected with WGS [70]. Using this approach, tumour fraction, along with SCNA amplitude, were shown to be correlated with disease progression and regression [44]. An increase in the tumour fraction relative to either the initial or previous aqueous humour sample was associated with disease progression, defined as an increase in vitreous seeding and/or increased or new tumour growth. In contrast, a stable or decreased tumour fraction was associated with a lack of active seeding or reduced tumour size. While this suggests that the analysis of the tumour fraction in aqueous humour-derived cfDNA could be used to monitor the therapeutic response, the authors note that the observed correlation is based on changes relative to previous samples from the same patient, and no overall tumour fraction threshold for the progression/regression in the disease state could be identified. Furthermore, a third of retinoblastoma patients lacked any SCNA. As a result, the tumour fraction could not be determined. The authors therefore suggest that a modified tumour fraction calculation based on *RB1* variants, which >99% patients carry, may be preferable.

Xu et al. [42] reported that the presence of a gain in chromosome 6p was also significantly correlated with seeding classification, whereby each increase in class from none to dust to sphere to cloud was associated with a two-fold increase in the presence of a gain in 6p. A 6p amplification was also associated with higher-risk histopathological features such as increased necrosis and choroidal invasion. To ascertain the minimal region gain (MRG), the authors compared the location of all chromosome 6p SCNAs detected. While the majority were found to span the entire 6p arm, the MRG was localised to a 19-Mbp region, which included the oncogene DEK.

A further major finding of the Berry group was that the genomic instability of cfDNA derived from aqueous humour is associated with the age at diagnosis [41,42]. Further investigation of this in their extended cohort of 50 patients identified a significant increase in retinoblastoma SCNAs in patients diagnosed after 12 months compared to those less than a year old [43]. Interestingly, unlike a previous publication [71], no significant difference in genomic stability was observed between hereditary and nonhereditary eyes. The observation of a discordant association between the age of onset or hereditary status and genomic instability was possible in the Berry patient cohort, as age of onset was not found to be significantly reduced in bilateral patents compared to those with unilateral disease. While this finding needs to be investigated further, it is supported by a comparative genomic hybridisation (CGH) study that reported significantly more chromosomal imbalances in unilateral retinoblastoma patients diagnosed after three years of age compared to those diagnosed before nine months [72]. Furthermore, the haploinsufficiency of *RB1* has been found insufficient to maintain genome stability [73]. Taken together, it could be hypothesised that it is, in fact, the timeframe between the first and second *RB1* hits that is correlated with genome stability, where a longer interval is associated with an increase in secondary genetic events.

### 3.2. Prognosis Using Plasma

As discussed earlier in this review, the possibility of a liquid biopsy using blood plasma may offer several advantages to the patient over using an aqueous humour sample. Yet, it remains questionable whether the genetic analysis of plasma-derived cfDNA would be able to match the high correlation shown between tumour DNA and cfDNA derived from the aqueous humour [38,40,41,42]. In an attempt to address this, specifically in relation to secondary somatic events, Berry et al. [45] compared SCNA profiling within plasma-derived cfDNA and cfDNA extracted from the aqueous humour of 20 eyes. While SCNAs were observed in 65% of the aqueous humour samples, none were observed within plasma cfDNA, suggesting that circulating tumour DNA in plasma is at too low a level for detection with the current technologies. It should be noted, however, that the volume of blood plasma used to extract cfDNA was matched to that of the aqueous humour volume at 100 μL. A significant advantage of using blood for a liquid biopsy is that a larger sample volume, often 10 mL of blood, equating to over 4 mL of plasma, is available. While taking this volume of blood may not be suitable for retinoblastoma patients, who are typically less than two years old, further investigation of the potential of plasma-derived cfDNA to detect secondary somatic events, using the maximum plasma volume available, is required.

Two studies have analysed cfDNA extracted from larger volumes of plasma in retinoblastoma patients with advanced or metastatic disease. Alongside performing *RB1* gene screening in cfDNA from 10 retinoblastoma patients with advanced disease (discussed in Section 2.2), Kothari et al. [47] undertook a targeted analysis of 13 *RB1* pathogenic SNVs previously identified in the paired tumour samples. Ten variants within eight patients were detected, suggesting that, in the future, the detection of the advanced disease state may be possible through the focused analysis of known pathogenic variants in plasma-derived cfDNA, although sensitivity is still questionable. The identification of patients whose disease has metastasised looks more encouraging, as the two highest *RB1* mutant allele frequencies observed in the plasma were in patients who went on to develop the metastatic disease. A further study of plasma-derived cfDNA of metastatic cancer patients was able to detect mutations in cancer driver genes within the blood of three retinoblastoma patients, indicating the possibility of using plasma for the analyses other secondary markers in metastatic disease [46]. Interestingly, relatively low levels of cfDNA were found in retinoblastoma patients compared to other cancers, such as cholangiocarcinoma, glioblastoma, and lung, where cfDNA loads were 200 times that seen in retinoblastoma patients, emphasising the difficulty in performing a liquid biopsy for retinoblastoma in the blood.

## 4. Discussion

The advances in molecular technology have enabled a proliferation of research into cell-free DNA in the last decade, with clinical utility and feasibility a significant focus. Two main applications, the liquid biopsy of cancer and noninvasive prenatal diagnosis for single-gene disorders, are of particular relevance to retinoblastoma. In this review, we summarised the exciting new research in this area.

The biopsy of a retinoblastoma tumour is not advocated due to the risk of extra-ocular spread. As a result, a nonhereditary diagnosis can only be confirmed if an eye is removed and, thus, somatic tissue is obtained as part of the patient’s treatment. Furthermore, the detection of secondary somatic events, which could have an influence on a patient’s prognosis, is not possible in vivo. By analysing cell-free DNA within the aqueous humour, the identification of somatic *RB1* mutations is now possible for patients undergoing conservative treatment. As well as providing a definitive diagnosis to nonhereditary patients, the identification of both apparently somatic-causal *RB1* mutations, followed by a targeted germline analysis, could also be of benefit to unilateral presenting patients who are germline mosaic but at a level too low to be detected by routine NGS screening (typically <5%). In adulthood, the offspring of these individuals would be offered testing on the identified somatic mutation, so that the potential consequence of occult mosaicism in the gonadal tissue is not missed.

The detection of foetal-derived cell-free DNA within maternal plasma now enables babies at risk of inheriting a *RB1* mutation to be diagnosed prior to birth without the risk of miscarriage. The likely future rise in families taking up this prenatal service will have a great impact on the planning of patient management and, potentially, patient outcomes [74]. Furthermore, an increase in the identification of the *RB1* gene status prior to delivery will facilitate robust studies into the potential visual and nonocular advantages of proposed early delivery compared to the risks of such an intervention. NIPD may also be of particular future benefit to retinoblastoma patients in middle- or low-income countries, where access to current pre- or postnatal testing may be limited [75], and is likely to present a significantly greater logistical challenge compared to a single maternal blood sample in the mid-trimester.

For the first time, the analysis of secondary genetic events within retinoblastoma tumours is possible in vivo. Shallow WGS of aqueous humour-derived cfDNA has identified several prognostic biomarker candidates, the most notable of which is a gain of chromosome 6p, which has also been implicated in several other nonretinal cancers [76]. Moreover, a recent publication has identified a higher prevalence of SCNAs, as well as *BCOR* alternations, in retinoblastoma patients with extraocular disease, further implicating them as markers of a more aggressive disease [77]. Prospective, longitudinal studies are now required to determine if the presence or absence of a gain in 6p or any other somatic copy number amplifications can predict the patient response and, therefore, in combination with other clinical characteristics, influence treatment decisions. *BCOR* and *CREBBP* should be included, along with *RB1,* in any future targeted sequencing in order to maximise the capture of recurrent non-*RB1* genetic variations within retinoblastoma patients. The variations commonly reported within these genes in retinoblastoma patients are limited to the exonic regions [11,12,77]; therefore, the coverage of these regions should be relatively straightforward, without a large impact on the sequencing capacity requirements.

The somatic profiles of tumours from different eyes of bilateral retinoblastoma patients have been found to be discordant, both in terms of the second somatic *RB1* pathogenic variant and non-*RB1* secondary events [12,78]. As a result, the genetic analysis of cfDNA derived from aqueous humour could provide an additional benefit to patients with bilateral disease, potentially allowing detailed prognostic information to be collected on each eye for a tailored treatment programme. A further advantage of the analysis of circulating tumour DNA could be the detection of clonal populations. Berry et al. [41] observed discordant tumour and cfDNA genetic profiles in retinoblastoma patients with multifocal tumours, and the idea that genetic variations in tumour-derived cfDNA reflects the clonal evolution of the corresponding tumour tissue has been well-investigated in other cancers [79].

An even greater refinement of precision treatment may become feasible with the identification of specific mutations and novel gene therapeutic approaches. These therapies would be delivered into the relatively closed compartment of the eye during the relatively short window of tumour development. Gene therapy approaches, such as enhancing the readthrough of nonsense mutations, might prove to be an effective prophylactic or salvage approach [80]. Nonsense mutations are the most common type of *RB1* gene germline mutation and, also, constitute a significant proportion of somatic mutations [7,8].

Several challenges still remain. The low levels of cell-free DNA within aqueous humour in patients undergoing treatment means that a genetic analysis—particularly, *RB1* mutation screening using capture-based NGS—is difficult. Earlier sampling through “diagnostic taps” plus improvements in library preparation and NGS technology will hopefully help address this issue. Nonetheless, the volume of sample available will always be a limitation with this sample type. Therefore, maximising the genetic information generated from a single aqueous humour aliquot will be important. A recent publication has reported a combined analysis strategy that enabled the identification of SCNAs through shallow WGS, followed by capture-based *RB1* mutation screening from the same aqueous humour sample [48].

The minimal levels of circulating tumour DNA within the blood, likely due to the closed compartment nature of the eye, means both a diagnosis and prognosis using plasma-derived cfDNA remains uncertain. The detection of the metastatic disease, following the identification of the causal *RB1* mutations in tumour-derived DNA or ocular cfDNA, appears to offer the most potential at present. At the current time, the routine detection of both hits in the *RB1* gene or non-*RB1* secondary somatic mutation screening using plasma will require improvements in molecular technology and/or postsequencing analysis. A significant area currently of interest in the liquid biopsy research community, which may also benefit retinoblastoma testing, is fragmentation analysis [81], which utilises the size differences between tumour and nonmalignant cfDNA to positively select tumour-derived cfDNA for subsequent analysis. A second option may be to explore a different sample type, such as cerebrospinal fluid, for the detection of CNS metastases.

## 5. Conclusions

The analysis of cell-free DNA from retinoblastoma patients within the ocular fluid or blood harnesses cutting edge genetic technologies and has the potential to transform the management of retinoblastoma across the clinical spectrum, from prenatal diagnosis to the late-malignant sequelae. While the authors accept that the publications described in this article are still relatively early studies, the unprecedented rate of development of new genomic and cell-free technologies, together with a desire for global collaboration in the retinoblastoma field, will hopefully mean that these findings can readily be confirmed, and patient benefits should rapidly be achieved. Furthermore, the dramatic fall in cost and increase in availability of genomic analyses, associated with the relative ease of sample transportation, means that delivery of these benefits to low- and middle-income countries should not be just a laudable aim but a deliverable expectation. Consequently, the full potential of cell-free DNA analyses on retinoblastoma patient management can be realised on a global scale.

## Figures and Tables

**Table 1:** Studies involving the analysis of cell-free DNA in retinoblastoma patients.

Study	Sample Type	Sample Numbers ^†^	NGS Technology	Targets	Analysis	Findings
Diagnosis						
Gerrish et al., 2019 [38]	AH	12|12|12	Targeted Capture	*RB1* & *MYCN*	SNV, CNV, LOH	100% *RB1* Mutation Detection
Gerrish et al., 2020 [39]	Maternal Plasma	15|NA|15	Amplicon based andTargeted Capture	*RB1*	SNV & RHDO	Prenatal Diagnosis 100% Concordant with Postnatal Diagnostic Result
Prognosis						
Berry et al., 2017 [40]	AH	6|3|3	WGS	NA	CNV	Correlation of cfDNA and tumour DNA CNV profiles
Berry et al., 2018 [41]	AH	63|29|26	WGS	NA	CNV	Correlation of SCNAs with Clinical Outcome
Xu et al., 2020 [42]	AH	116|50|46	WGS	NA	CNV	Chr 6p amplification associated with treatment outcome
Polski et al., 2020 [43]	AH	54|54|50	WGS	NA	CNV	Correlation of Genome Stability with Age of Onset of RB
Polski et al., 2020 [44]	AH	78|20|20	WGS	NA	CNV	Correlation of Tumour Fraction and Therapeutic Response
Berry et al., 2020 [45]	AH Plasma	20|20|17 17|NA|17	WGS	NA	CNV	CNVs detected within AH samples but not within plasma
Palmieri et al., 2020 [46]	Plasma	3|NA|3 **	Targeted Capture	Oncomine ^(TM)^ Pan Cancer Cell-Free Assay	SNV, CNV	Detection of Secondary Somatic Events
Combined Prognosis and Diagnosis						
Kothari et al., 2020 [47]	Plasma	10|NA|10	Targeted Capture	MSK-IMAPCT Panel	SNV *	54% de novo *RB1* Mutation Detection 77% Targeted *RB1* Mutation Detection
Xu et al., 2020 [48]	AH	7|7|6	WGS & Targeted Capture	NA *RB1* & *MYCN*	CNV, SNV, CNV, and LOH	Combined SCNA Detection and *RB1* Mutation Detection

^†^ Sample numbers given, separated by vertical bars, refer to the number of individual cell-free (cf)DNA samples from|Eyes|Patients analysed by each study. AH: aqueous humour. NA: not applicable. RHDO: relative haplotype dosage. SNV: single-nucleotide variation. CNV: copy number variation. LOH: loss of heterozygosity. SCNA: somatic copy number alteration. RB: retinoblastoma. NGS: next-generation sequencing. WGS: whole-genome sequencing. * Only SNVs reported. ** Retinoblastoma patients only.

## Data Availability

No new data were created or analysed in this study. Data sharing is not applicable to this article.
